# Waist-to-Height Ratio (WHtR) in Predicting Coronary Artery Disease Compared to Body Mass Index and Waist Circumference in a Single Center from Saudi Arabia

**DOI:** 10.1155/2020/4250793

**Published:** 2020-03-16

**Authors:** Mostafa Q. Alshamiri, Faisal Mohd A Habbab, Saad Saeed AL-Qahtani, Khalil Abdullah Alghalayini, Omar Mohammed Al-Qattan, Fayez El-shaer

**Affiliations:** College of Medicine, King Saud University, Riyadh, Saudi Arabia

## Abstract

This study aims to study the efficiency of the Waist-to-Height Ratio (WHtR) for determining coronary artery disease. It compares the frequency of abnormal WHtR, as a proxy for abdominal obesity, to that of body mass index (BMI) and waist circumference (WC). It also relates the findings to other cardiometabolic risk factors in University Hospital patients. A cross-sectional study design was used, where a sample of 200 patients (142 males and 58 females) who attended the adult cardiac clinic were purposively included. BMI, WC, and WHtR were measured, where frequencies of WHtR were compared to those of BMI and WC. The findings were related to the history of coronary artery disease (CAD) and history of cardiometabolic risk factors, including diabetes mellitus (DM), hypertension (HTN), and hyperlipidemia. Majority of the male patients were older, taller, and had a lower BMI value. It also showed that the prevalence of dyslipidemia and CAD was higher in male patients. No significant difference between both genders was noticed for weight, WC, WHtR, hypertension, or DM. BMI was least associated with high-risk cardiac population in both males and females (39.4% and 60.3%), followed by WC (84.5% and 96.6%, respectively). WHtR showed the highest association with gender (male 98.6% and females 98.3%). These findings were noticed in patients with all risk factors. WHtR is superior to BMI and WC for determining the elevated risk of diabetes, hypertension, dyslipidemia, and CAD in a single university institute. The role of WHtR in both normal and diseased Saudi population should be delineated.

## 1. Introduction

The prevalence of cardiovascular disease (CVD) is on rise, given the increased incidence of obesity and lifestyle changes [1]. The World Health Organization (WHO) reports that about two billion of the population suffers from obesity [2]. Elsami et al. [3] reported that obesity account for the major deaths among CVD patients annually, which is estimated to reach about 23.6 million by 2030. This calls for the attention of the health professionals to seek modalities that help identify the obesity cases and prevent the occurrence of its adverse outcomes.

A piece of overwhelming scientific evidence and reference to WHO, body mass index (BMI), is identified as the index for characterizing obesity, which is associated with various adverse health risks [4]. Although BMI has been used as a proxy for obesity for a long time, it does not differentiate between the muscular mass and the overweight, except at high BMIs. It characterizes the total fat in the body but cannot distinguish between individuals with different types of fat distribution [5].

People with a “central” type of fat are at a greater health risk than those whose fat is distributed. There has been general agreement that health risks, predominantly CVD and diabetes, can be efficiently determined by the relative distribution of the excess fat as compared to its total amount [6]. The use of imaging techniques such as computed tomography (CT) [7] and magnetic resonance imaging (MRI) [8] has subsequently indicated that the “unhealthy apple shape” is characterized by a preferential deposition of fat in the internal visceral fat rather than in the external subcutaneous fat, which leads to the “healthy pear shape”. Several indices, such as waist circumference (WC) and the waist to hip ratio (WHtR), are proposed to assess the visceral fat [9]. However, both indices are known for their overestimation or underestimation of cardiometabolic risk in short- and tall-heighted people, whose values are required to be adjusted across different races [10].

The ratio (R) of the waist circumference- (WC-) to-height (Ht), called WHtR, was originally proposed simultaneously in Japan [8] and the UK [11–14] to assess the body shape and monitor the reduction of risk. Both suggested that WHtR values above 0.5 indicate an increased health risk. It is believed that a simple index such as WHtR is a good proxy for central obesity and has substantial practical advantages. The greater propensity for South Asians to develop diabetes at lower BMI than white Europeans has been recognized, leading to different BMI ranges being suggested for South Asians [15]. The use of WHtR avoids such concern because the adjustment of waist circumference for height means that the same boundary values are suitable for both ethnic groups. Therefore, WHtR represents a rapid and effective global indicator for health risks of obesity, and its use could simplify the international public health message [16–18]. Most studies show that obese individuals with high WHtR are more vulnerable to CVD diseases [18, 19]. Son [20] and Ashwell [21] showed WHtR cutoff value of ≥0.5 as efficient and supported that increased adiposity was substantially related to the risk of CVD. However, these cutoff values have been established for Asian populations and none for the non-Asian populations [22, 23], particularly in Saudi Arabia [22, 23], where its cutoff value may differ. Also, WHtR has not been studied among the Saudi population, which further drives this research. Thereby, to bridge this gap, this study examines the WHtR capability in predicting coronary artery disease. It compares the reliability of abnormal WHtR, as a proxy for abdominal obesity, with BMI and WC and relates the findings to other cardiometabolic risk factors in adults.

## 2. Materials and Methods

### 2.1. Study Design

A cross-sectional study design was used for comparison of the reliability of abnormal WHtR for BMI and WC. The selection of the cross-sectional study design is based on its use by the previous research, which found it effective for determining the effective predictors of the disease (such as coronary heart disease, hypertension, and more) [24–27]. Accordingly, the present study follows a case report form. The rationale for using this design was also based on its integration of the protocols.

### 2.2. Sample

**Figure 1:**
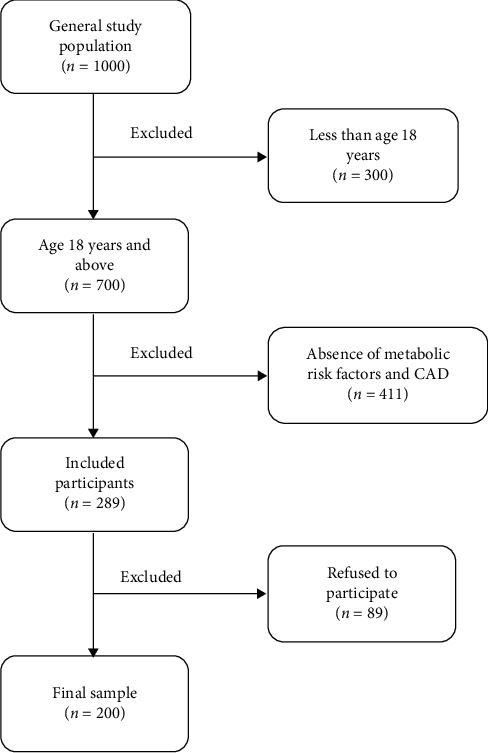
Flowchart for participants selection.

The study sample constitutes (200) adult cardiac patients who attended the single cardiology outpatient clinic (Medical City King Saud University) in Saudi Arabia. This sample was purposively recruited from the population of 1000 based on the determined inclusion criteria. The study population was small due to the inclusion of the single centre and the followed inclusion and exclusion criteria. The selection of the sample was also based on the study in [24], which found effective results on the small sample size. Keeping it as the base, the sample of 200 participants was selected. Also, the inclusion criteria required participants to be cardiac patients aged 18 years or above with metabolic risk factors and CAD. This criterion was determined as these patients had abnormal central obesity, which is an indicator of increased health risk for cardiac disease. Prior to recruiting the participants, ethical approval was achieved from the Institutional Ethical Board at the college of medicine at King Saud University. The researcher also obtained a signed consent form from the study participants. The interviewer informed all the participants about the purpose of this noninterventional study, their role, all potential risks, benefits, and their right to refuse participation.  Figure 1 presents the selection of the final study sample.

### 2.3. Data Collection

Participants' demographic details were collected, including data concerning patient's age, gender, weight, height, WC, history of coronary artery disease (CAD) and history of cardiometabolic risk factors including diabetes mellitus (DM), hypertension (HTN), and hyperlipidemia. BMI was measured through the weight and height of the participant [28]. WC was measured using a flexible nonstretch tape to the nearest 0.1 cm at midpoint between the lower rib and the iliac crest while subjects were standing and breathing normally [29]. WC was used in statistical analyses. Abdominal obesity indicators were WC and WHtR. The population-specific WC cutoff points were determined in the present study, along with the generic ones for abdominal obesity in metabolic syndrome definition (WC ≥94 cm in men and ≥80 cm in women) [30]. WHtR was calculated through the waist circumference ratio to the height. The standard cutoff points of 0.5 were used for WHtR. The method, as described in [16], was used for its evaluation, which did not calculate the WHtR due to the difference in the ethnic backgrounds, but supported 0.5 as the cutoff point. The value of 0.5 was used, which denotes to keep the WC less than half of the height, and also provides the first boundary value for increased risk on public health tool, i.e., a WC against the height chart [16]. Cardiometabolic risk factors, as well as the presence of coronary artery disease, were noted for all patients.

### 2.4. Data Analysis

Data were analysed using IBM SPSS (Statistical Package for Social Sciences) version (Pc + 21.0). Descriptive statistics (frequencies, percentages, mean, and standard deviation) and *χ*^2^ test were used to describe the categorical variables, while student's *t*-test was used for continuous variables. The significance value (*p* value) of <0.05 and 95% confidence intervals (CI) were used to report the precision of results.

## 3. Results and Discussion

Among 200 participants, 142 were males, and 58 were females. It showed that many patients were exposed to metabolic risk factors such as hypertension, diabetes mellitus, and/or dyslipidemia (reaching up to 69% in males and 62% in females). It showed that the majority of the patients visiting the clinic had CAD (about 72% in males and 41% in females). The baseline characteristics of the participants are presented in Table 1. Comparison of patients' characteristics in terms of gender showed that male patients were older as compared to female patients (62.58 ± 11.84 vs. 58.38 ± 11.75 years, *p*=0.024), taller (165.70 ± 7.78 vs. 155.20 ± 6.17 cm, *p*=<0.001), and had lower BMI value (28.31 ± 4.78 vs. 32.57 ± 6.41 kg/m^2^, *p*=<0.001). It also shows high prevalence of dyslipidemia in males as compared to females (62.68% vs. 43.10%, *p*=0.012). Similarly, CAD was higher in males (72.54% vs. 41.38%, *p*=<0.001). Also, no significant statistical difference was found among males and females in terms of weight, WC, WHtR, hypertension, and diabetes mellitus.

Table 2 shows the result of three indices among participants. It showed that BMI was least associated with high-risk cardiac population for both genders, i.e., males and females (39.4% vs. 60.3%), *p*=0.0163 followed by WC (84.5% vs. 96.6%, *p*=0.008), respectively. The highest association was found for WHtR, which was 98.6% for males and 98.3% for females (*p* value 0.4969), indicating it as a reliable indicator for CAD risk than the other two, particularly more than BMI.

Table 3 shows the frequency for positive and negative results of WC, BMI, and WHtR in relation to risk factors (HTN, DM, and dyslipidemia) and CAD.

Table 4 shows that WHtR has the highest sensitivity for the three risk factors (98.51%, 99.10 and 99.12, i.e., HTN, DM, and dyslipidemia, respectively), where it reached 100% for CAD. It also had the highest negative predictive value (NPV) for dyslipidemia and CAD (66.66 and 66.66, respectively) when compared to WC and BMI. The WC was associated with the highest positive predicted value (PPV), except for dyslipidemia. Although BMI had higher specificity for the three risk factors, i.e., CAD than WC and WHtR, it was associated with the lowest sensitivity, NPV, and PPV. The calculation of the sensitivity and specificity for the predictive value was based on the research of Haun et al. [25], which used a similar test for predicting high coronary risk.

To the best of the author's knowledge, this study is the first to be conducted on the Saudi population. Using a cross-sectional design, it showed that WHtR >0.5 was superior to BMI and WC in identifying men and women at elevated risk of diabetes, hypertension, dyslipidemia, and CAD. Studies in different age groups have shown that aging leads to the redistribution of adipose tissue and internalization of abdominal fat, especially in women [31, 32]. Accumulation of fat tissue, especially in the abdominal region, predisposes to a series of risk factors through a highly frequent association with outcomes that favour the occurrence of cardiometabolic disorders [33, 34]. Such changes in body composition with aging could alter the cutoff points for measures such as BMI and WC, where WHtR is a potentially advantageous measure due to its adjustment by height [35], thus justifying a single reference value independent of age and gender [36]. The current study verified such evidence, as WHtR was superior to BMI and WC in identifying the risk of cardiometabolic disorders. This is contradicting the findings of Li et al. [37], which showed BMI as a better predictor for cardiovascular disease than WHtR.

Although BMI does not measure body composition, it does have a good diagnostic potential for nutritional status in epidemiological studies, with a weak correlation with height and strong correlation with absolute fat mass. High BMI is positively associated with morbidity and mortality from various chronic noncommunicable diseases [38, 39]. However, for better diagnosis of overweight, studies recommend that BMI values be combined with other measures of adiposity such as WC, in individual and collective assessments, which help to make better health prediction using these adiposity indicators [40]. The findings of the study recommend that health professionals should look beyond BMI, which is not enough to assess early risk, and fails to classify a considerable portion of the population at imminent risk. These results are consistent with other research studies on cardiovascular risk factors [41].

In the current study, BMI was the least to be associated with high-risk cardiac population, in both males and females as compared to WC and WHtR, as BMI had the lowest values for highlighting the cardiometabolic risk and the lowest sensitivity, specificity, PPV, and NPV for CAD. The highest value was found for WHtR, which is corroborated by another study's finding [34]. WHtR has been viewed as a simple primary risk assessment tool that identifies more subjects at “cardiometabolic risk” than the combination of BMI and WC. Therefore, researchers are recommended to replace the combination of BMI and WC by the routine use of WHtR since individuals with high WC are being classified in the healthy BMI range, thus overlooking a large group at potential risk [42].

The 0.50 cutoff point for WHtR in various populations was proposed in a systematic review as the best value for both genders, different age groups (children, adolescents, and adults), and different ethnic groups [36]. Therefore, it is advised that everyone keep their waist circumference less than half their height [37]. WHtR proposed to keep values below 0.50 as low-risk to health, 0.5 to 0.6 as suggestive of risk, and greater than 0.60 as high-risk [43]. Also, disease prevention and health recovery measures should be recommended for values above 0.50 [43]. This study found that mean WHtR of 0.50 was indicative of elevated risk cardiometabolic disorders.

These findings establish the efficacy of the WHtR as a screening tool. It recommends that intensive lifestyle modification should be introduced for reducing the waist circumference, such as the adaptation of healthy eating habits and exercise. However, the present study has several potential limitations. First, this is a cross-sectional design study, with its inherent limited interpretation of cause-and-effect temporality. Second, it included a small number of cardiac patients. Third, it is a single-centre study. The fourth limitation includes it being a study that included patients who were already diagnosed as CAD, and they were on medication; hence, the relationship of BMI, WC, and WHtR at the time of diagnosis was not known. Therefore, further studies are recommended to expand and delineate the role of WHtR as a predictive tool of cardiometabolic risk, as well as its association with CVD in both normal and diseased Saudi population.

## 4. Conclusion

WHtR is a simple and effective index of CAD and cardiometabolic risk among male and female cardiac patients, which can be superior to BMI and WC. WHtR of >0.5 clearly identifies 251 men and women at an elevated risk of CAD. The results of this study are beneficial for the health-care experts and professionals for reducing the risk of cardiometabolic disorders among patients. However, the generalizability of the study should be carefully considered, given its small sample size and a single centre study. A study with improved sample size and across various centres is needed for clarifying the role of WHtR, as well as its relation to cardiometabolic risk factors and CVD in both normal and diseased Saudi population.

## Figures and Tables

**Table 1:** Characteristics of participants.

	Men (*n* = 142)	Women (*n* = 58)	*p* value
Age (yrs.) (mean ± SD)	62.58 ± 11.84	58.38 ± 11.75	0.024^*∗*^
Weight (kg) (mean ± SD)	77.92 ± 15.02	78.52 ± 16.63	0.810
WC (cm) (mean ± SD)	106.57 ± 11.64	104.55 ± 13.37	0.318
Height (cm) (mean ± SD)	165.70 ± 7.78	155.20 ± 6.17	<0.001^*∗*^
BMI (kg/m^2^) (mean ± SD)	28.31 ± 4.78	32.57 ± 6.41	<0.001^*∗*^
WHtR	0.64 ± 0.07	0.67 ± 0.09	0.730
Hypertension, *n* (%)	98 (69.01)	36 (62.07)	0.408
Diabetes mellitus, *n* (%)	82 (57.75)	29 (50.00)	0.349
Dyslipidemia, *n* (%)	89 (62.68)	25 (43.10)	0.012^*∗*^
CAD, *n* (%)	103 (72.54)	24 (41.38)	<0.001^*∗*^

^*∗*^Significant difference between men and women, *p* < 0.05.

**Table 2:** Abnormal values of study variables in males and females.

	Men (*n* = 142)	Women (*n* = 58)	*p* value
WC *n* (%)			0.0163^*∗*^
≥94 cm (male) ≥80 cm female)	120 (84.5%)	56 (96.6%)	
BMI *n* (%)			0.008^*∗*^
≥30 (kg/m^2^)	56 (39.4%)	35 (60.3%)	
WHtR *n* (%)			0.4969
≥0.5	141 (98.6%)	57 (98.3%)	

^*∗*^Significant difference between men and women, *p* < 0.05.

**Table 3:** Positive and negative results as per risk factors and CAD.

Study variables		HTN		DM		Dyslipidemia		CAD	
		(−)	(+)	(−)	(+)	(−)	(+)	(−)	(+)
		*n* (%)	*n* (%)	*n* (%)	*n* (%)	*n* (%)	*n* (%)	*n* (%)	*n* (%)
WC	<80 cm (W) <94 cm (M)	12 (18.2%)	12 (9.0%)	16 (18.0%)	8 (7.2%)	12 (14.0%)	12 (10.5%)	7 (9.6%)	17 (13.4%)
	≥80 cm (W) ≥94 cm (M)	54 (81.8%)	122 (91.0%)	73 (82.0%)	103 (92.8%)	74 (86.0%)	102 (89.5%)	66 (90.4%)	110 (86.6%)
*p* value		0.067		0.027^*∗*^		0.514		0.503	
BMI	<30 (kg/m^2^)	44 (66.7%)	65 (48.5%)	49 (55.1%)	60 (54.1%)	44 (51.2%)	65 (57.0%)	39 (53.4%)	70 (55.1%)
	≥30 (kg/m^2^)	22 (33.3%)	69 (51.5%)	40 (44.9%)	51 (45.9%)	42 (48.8%)	49 (43.0%)	34 (46.6%)	57 (44.9%)
*p* value		0.016		1.000		0.474		0.883	
WHtR	<0.5	1 (1.5%)	2 (1.5%)	2 (2.2%)	1 (0.9%)	2 (2.3%)	1 (0.9%)	3 (4.1%)	0 (0.0%)
	≥0.5	65 (98.5%)	132 (98.5%)	87 (97.8%)	110 (99.1%)	84 (97.7%)	113 (99.1%)	70 (95.9%)	127 (100.0%)
*p* value		1.000		0.586		0.578		0.047^*∗*^	

**Table 4:** Sensitivity, specificity, positive predictive value, and negative predictive value of risk factors and CAD.

		HTN (%)	DM (%)	Dyslipidemia (%)	CAD (%)
WC	(i) Sensitivity	91.05	92.79	89.47	63.50
	(ii) Specificity	18.18	17.98	13.95	9.59
	(iii) Positive predictive value	69.32	59.52	50.00	86.64
	(iv) Negative predictive value	50.00	66.66	57.96	29.17

BMI	(i) Sensitivity	51.49	45.95	42.98	44.88
	(ii) Specificity	44.66	55.06	51.16	53.42
	(iii) Positive predictive value	56.04	56.04	53.84	62.64
	(iv) Negative predictive value	44.95	44.94	40.37	35.78

WHtR	(i) Sensitivity	98.51	99.10	99.12	100.00
	(ii) Specificity	1.51	2.25	2.32	4.11
	(iii) Positive predictive value	68.02	55.83	57.36	64.46
	(iv) Negative predictive value	33.33	33.33	66.66	66.66

## Data Availability

The data used to support the findings of this study are available from the corresponding author upon request.
